# Dynamics of Bacterioplankton Communities during Wet and Dry Seasons in the Danjiangkou Reservoir in Hubei, China

**DOI:** 10.3390/life13051206

**Published:** 2023-05-17

**Authors:** Qing Yang, Dewang Li, Wei Chen, Liming Zhu, Xi Zou, Lian Hu, Yujie Yuan, Shan He, Fang Shi

**Affiliations:** Institute of Hydroecology, Ministry of Water Resources & Chinese Academy of Sciences, Wuhan 430079, China

**Keywords:** bacterioplankton diversity, 16S rRNA gene, spatiotemporal variations, wet and dry season, Danjiangkou Reservoir

## Abstract

Water quality is directly linked to drinking water safety for millions of people receiving the water. The Danjiangkou Reservoir is the main water source for the Middle Route of the South-to-North Water Diversion Project (MR-SNWDP), located in the vicinity of Henan and Hubei provinces in China. Aquatic microorganisms are key indicators of biologically assessing and monitoring the water quality of the reservoir as they are sensitive to environmental and water quality changes. This study aimed to investigate the spatiotemporal variations in bacterioplankton communities during wet (April) and dry (October) seasons at eight monitoring points in Hanku reservoir and five monitoring points in Danku reservoir. Each time point had three replicates, labeled as wet season Hanku (WH), wet season Danku (WD), dry season Hanku (DH), and dry season Danku (DD) of Danjiangkou Reservoir in 2021. High-throughput sequencing (Illumina PE250) of the 16S rRNA gene was performed, and alpha (ACE and Shannon) and beta (PCoA and NDMS) diversity indices were analyzed. The results showed that the dry season (DH and DD) had more diverse bacterioplankton communities compared to the wet season (WH and WD). Proteobacteria, Actinobacteria, and Firmicutes were the most abundant phyla, and Acinetobacter, Exiguobacterium, and Planomicrobium were abundant in the wet season, while polynucleobacter was abundant in the dry season. The functional prediction of metabolic pathways revealed six major functions including carbohydrate metabolism, membrane transport, amino acid metabolism, signal transduction, and energy metabolism. Redundancy analysis showed that environmental parameters greatly affected bacterioplankton diversity during the dry season compared to the wet season. The findings suggest that seasonality has a significant impact on bacterioplankton communities, and the dry season has more diverse communities influenced by environmental parameters. Further, the relatively high abundance of certain bacteria such as Acinetobacter deteriorated the water quality during the wet season compared to the dry season. Our findings have significant implications for water resource management in China, and other countries facing similar challenges. However, further investigations are required to elucidate the role of environmental parameters in influencing bacterioplankton diversity in order to devise potential strategies for improving water quality management in the reservoir.

## 1. Introduction

High-quality and plentiful water is necessary for the survival of human society and to sustain the viability of ecosystems [1]. It is crucial to comprehend how human interventions and other natural phenomena have imposed detrimental environmental problems such as water scarcity and a decline in water quality [2]. In China, water resources are unevenly distributed, there being a surplus of water in the south and a water shortage in the north [3]. The Middle Route of the South-to-North Water Diversion Project (MR-SNWDP), which represents the world’s longest water diversion project, was introduced in December 2014 to address the severe water scarcity in northern China [4,5]. The Danjiangkou Reservoir (DJR) is the primary water source for the MR-SNWDP, and its normal water storage level has been raised from 157 m to 170 m by increasing its height. The new inundation area was expanded by 302.5 km^2^. Since its introduction, the MR-SNWDP has diverted 40 billion cubic meters of water supply to the Hebei and Henan provinces [6]. Its water quality is directly linked to drinking water safety for millions of people receiving the water. Therefore, it is of utmost importance to conduct regular monitoring of the water quality in the DJR [7,8]. As a result, the DJR ecosystem is being rebuilt, making it a great place to research the microbial community’s diversity, interaction, and function in major reservoirs [9].

Aquatic microorganisms are the main constituents of the aquatic community and play a crucial role in various biogeochemical processes, such as the movement of matter in aquatic habitats and the regulation of chemical cycles throughout the ecosystem. Aquatic microorganisms are key indicators of biological assessment and for monitoring reservoir water quality as they are sensitive to environmental and water quality changes [10,11,12]. Therefore, studies of the community structure, function of aquatic microorganisms, and how they respond differently to physical and chemical parameters are of immense importance for the ecological management and maintenance of the aquatic environment [13]. Microbial communities in the aquatic environments can be influenced by environmental factors such as water-dissolved oxygen (DO), pH, temperature, and the availability of phosphorus and nitrogen nutrients, as well as by biotic factors such as predation and competition [14].

Currently, there are limited studies available on the bacterioplankton community structure and associated environmental factors in the DJR [15]. Advanced culture-free molecular biology approaches have emerged as an essential strategy for investigating microbial diversity because a large number of microorganisms are non-culturable. Recently, the high-throughput sequencing (HTS) technique has provided an excellent strategy to study the distribution, composition, and influencing factors of the bacterioplankton communities [16,17]. A previous study using HTS was conducted to determine the bacterioplankton community composition along with influencing factors in the DJR. The results showed that the bacterioplankton community consisted of 27 phyla and 336 genera. It was also found that TN, pH, and COD can dramatically affect the composition of the bacterioplankton community [18]. At the DJR, the wet season lasts from August to January and the dry season is from February to July of the subsequent year. In the DJR, bacterioplankton can fluctuate annually and seasonally, although there is as yet no thorough comparative research across different years and seasons [6]. The most significant factor affecting water quality in the DJR is TN, which is generally high in nitrogen and phosphorus. The bacterioplankton community is a primary contributor to P and N cycles [19]. 

In the current study, a high-throughput sequencing platform (Illumina PE250) was used to characterize the 16S rRNA gene (V4 region) of the filter membrane microorganisms in Danjiangkou Reservoir water samples in April (high-water or wet season) and October (low-water or dry season) in 2021. This study mainly focused on the bacterioplankton diversity, their functions, and relationships with the environmental factors of the Danjiangkou Reservoir water body during the wet and dry seasons at different monitoring points in Hanku and Danku reservoirs.

## 2. Materials and Methods

### 2.1. Study Area and Sample Collection

**Figure 1:**
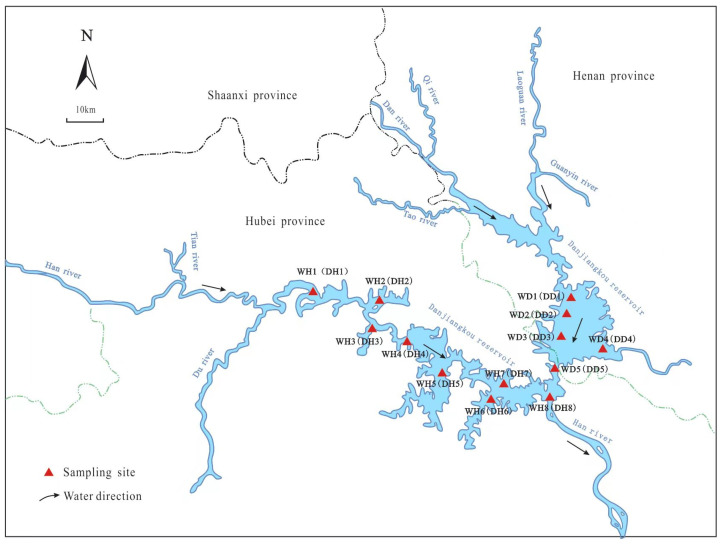
Sampling site locations in Danjiangkou Reservoir: a total of 13 sampling sites (8 sampling sites at Hanku reservoir in wet (WH) and dry (DH) seasons each: WH1 and DH1, WH2 and DH2, WH3 and DH3, WH4 and DH4, WH5 and DH5, WH6 and DH6, WH7 and DH7, and WH8 and DH8; 5 sampling sites at Danku reservoir in wet (WD) and dry (DD) seasons each: WD1 and DD1, WD2 and DD2, WD3 and DD3, WD4 and DD4, and WD5 and DD5).

The Danjiangkou Reservoir (32°36′–33°48′ N, 110°54′–111°48′ E) is the main water source for the MR-SNWDP (Middle Route of the South-to-North Water Diversion Project), located in the vicinity of Henan and Hubei provinces in China [20]. The reservoir is linked to the north subtropical climatic zone, with exceptional climatic variations. It experiences an average temperature range from 14.6 to 21.2 °C, and precipitation from 542 to 1173 mm annually [21]. This study was designed to compare bacterioplankton diversity between wet and dry seasons at monitoring points locations in Hanku and Danku reservoirs labeled as wet season Hanku: WH; wet season Danku: WD; dry season Hanku: DH; and dry season Danku: DD of Danjiangkou Reservoir. A total of 13 sampling points (8 sampling points from Hanku reservoir and 5 sampling points from Danku reservoir) were selected in the Danjiangkou Reservoir for sample collection during the high-water or wet season (April) and the low-water or dry season (October) in 2021. The location of sampling sites is presented in Figure 1. At each sampling point, three replicates for each sample were collected from the surface, shallow, and deeper regions of the reservoir. There were 26 samples (13 samples from each season) in total for measuring the water parameters and bacterioplankton diversity after combining the triplicate samples into one sample for each site. Detailed, labeled sampling information is given in Table S1. These water samples were processed further for the DNA extraction of microorganisms, as well as the physicochemical analysis of reservoir water quality. For the analysis of bacterioplankton communities, water samples were stored in polyethylene bottles (thermo-boxes) at 4 °C and kept cool while they were transported to the laboratory. A filtration membrane was used to collect membrane microorganisms by filtering 1000 mL of each water sample. The microbial cells were collected within twenty-four hours after sampling, and filters with microbial cells were stored at −20 °C for later molecular analysis. At the same time, the physicochemical parameters were measured based on the environmental quality standard of surface water in China.

### 2.2. Physicochemical Parameters

In the current study, thirteen physicochemical parameters, including optical dissolved oxygen (ODO), temperature that was measured in situ using a YSI 6920 Sonde (YSI Inc., Yellow Springs, OH, USA), and other parameters including turbidity (turd), pH, ammonia nitrogen (NH_4_-N), nitrate (NO_3_^−^), nitrous nitrogen (NO3-N), total nitrogen (TN), phosphate (P), total phosphate (TP), chlorophyll (Chl), and permanganate (COD_Mn_), were determined at each sampling site during the wet and dry season (DH, DD, WH, and WD) according to the National Drinking Water Quality Standard protocol (GB5749-2006).

### 2.3. DNA Extraction, PCR Amplification, and Illumina NovaSeq Sequencing

The next-generation sequencing technique has significantly increased the studies of microbial diversity and ecology by employing samples from various environments such as water and soil [22,23]. Microbial DNA was extracted from the membrane filters using the E.Z.N.A.^®^ Water DNA Kit (OMEGA Bio Tek, Norcross, GA, USA) following the supplier’s instructions. In PCR, specific primers 338F (ACTCCTACGGGAGGCAGCA) and 806R (ACTCCTACGGGAGGCAGCA) were used for the amplification of the V4 region of the 16S ribosomal RNA (rRNA) gene [24]. Amplicons were then isolated on 2% agarose gel and purified with a DNA Gel Extraction Kit (Axygen Biosciences, Union City, CA, USA) following the manufacturer’s guidelines. Then, amplicons were quantified by QuantiFluor™-ST (Promega, Madison, WI, USA) and Illumina PE250 sequencing was applied for hands-on sequencing to identify bacterioplankton diversity in the Danjiangkou Reservoir. 

### 2.4. Data Analyses

The Illumina PE250 sequencing data were then processed and quality filtered through QIIME (version 1.17) [25]. The bacterial sequences were detected and subsequently clustered into operational taxonomic units (OTUs) with 97% similarity using UPARSE (version 7.1 Tiburon, CA, USA). This OTU number represents the richness of species for each sample. Further, UCHIME was used to identify and remove the chimeric sequences. The alpha diversity indices (Ace and Shannon) were estimated based on OUTs (97% similarity) using MOTHUR (version 1.30, https://www.mothur.org/ accessed on 10 October 2022) [26]. Further in beta diversity, non-metric multidimensional scaling (NDMS) and principal coordinate analysis (PCoA) were performed using QIIME software (Version 1.9.1) to evaluate the differences in spatial and temporal patterns of the microbial community for all samples during the dry and wet seasons. Further, the Kruskal–Wallis H test was used to assess the significant differences in bacterioplankton communities in all samples. The functional prediction profiles of 16S gene data were explored using the Tax4fun software package in R software. A redundancy discriminate analysis (RDA) was used to find the correlation of environmental factors with the diversity of the bacterioplankton community using the vegan package in R software. 

## 3. Results

### 3.1. High-Throughput Sequencing Data Annotation Results

High-throughput sequencing of the 16S rRNA gene revealed the bacterioplankton communities’ composition in water samples collected during wet and dry seasons from Hanku (WH and DH) and Danku (WD and DD) sampling points in the Danjiangkou Reservoir. The sequencing data yielded a total of 8,432,783 clean reads (with an average of 108,112) after quality filtering the raw reads from the 8,526,975 (with an average of 109,320). These reads were clustered into a total of 4254 operational taxonomic units (OTUs) using the Silva database (release132) (Figure S1). Further, 136 OTUs were common among WH, DH, WD, and DD, while 66, 2024, 88, and 269 OTUs were unique to WH, DH, WD, and DD, respectively. Moreover, the rarefaction and rank abundance curves were plotted, which showed that the number of OTUs was adequate for each sample to represent the diversity of bacterioplankton communities in WH, DH, WD, and DD samples (Figure S2A,B). Dry season samples (DH and DD) had more richness and evenness than wet season samples (WH and WD).

### 3.2. Alpha Diversity

**Figure 2:**
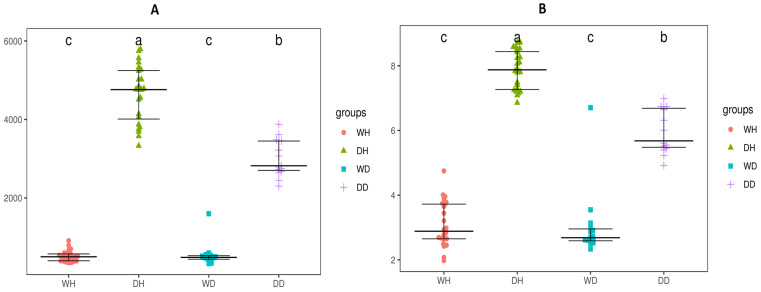
Alpha diversity indices (**A**) ACE and (**B**) Shannon showing the significant (*p* < 0.05) bacterioplankton communities’ differences between WH, WD, DH, and DD samples of the Danjiangkou reservoir (8 sampling sites at Hanku reservoir in wet season (WH): WH1, WH2, WH3, WH4, WH5, WH6, WH7, and WH8; 8 sampling sites at Hanku reservoir in dry season (DH): DH1, DH2, DH3, DH4, DH5, DH6, DH7, and DH8; 5 sampling sites at Danku reservoir in wet season (WD): WD1, WD2, WD3, WD4, and WD5; and 5 sampling sites at Danku reservoir in dry season (DD): DD1, DD2, DD3, DD4, and DD5). Letters a to c in figure shows significant differences between different groups.

The bacterioplankton communities’ abundance and diversity were analyzed spatiotemporally in wet and dry seasons at Hanku and Danku sampling points using ACE and Shannon indices (Figure 2A,B). The ACE index (community abundance index) showed that the richness of bacterioplankton communities differed significantly (*p* < 0.05) seasonally and was higher (*p* < 0.05) during the dry season than the wet season (DH > WH and DD > WD) (Figure 2A). Spatially, there was a significant difference (*p* < 0.05) at both sampling sites (DH vs. DD) during the dry season, whereas no significant (*p* > 0.05) difference was observed during the wet season at both sampling sites (WH vs. WD). The Shannon index (community diversity index) also revealed significantly higher (*p* < 0.05) bacterioplankton diversity during the dry season than wet season (DH > WH and DD > WD) (Figure 2B). Spatially, the community diversity index revealed there was a significant difference (*p* < 0.05) at both sampling sites (DH vs. DD) during the dry season, whereas no significant (*p* > 0.05) difference was observed during the wet season at both sampling sites (WH vs. WD).

### 3.3. Differential Bacterial Communities

**Figure 3:**
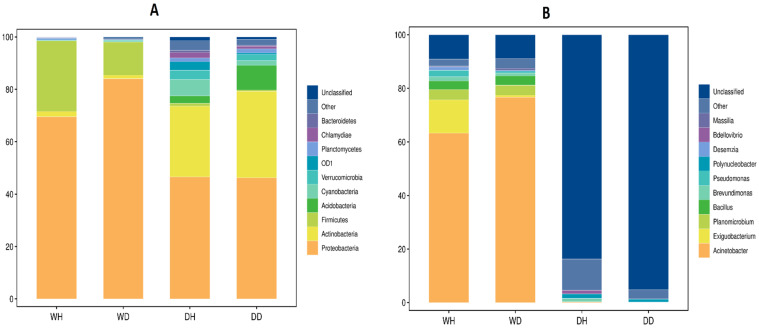
Relative abundance of dominant bacterioplankton communities (**A**) phyla and (**B**) genera of WH, WD, DH, and DD samples of the Danjiankou reservoir (8 sampling sites at Hanku reservoir in wet season (WH): WH1, WH2, WH3, WH4, WH5, WH6, WH7, and WH8; 8 sampling sites at Hanku reservoir in dry season (DH): DH1, DH2, DH3, DH4, DH5, DH6, DH7, and DH8; 5 sampling sites at Danku reservoir in wet season (WD): WD1, WD2, WD3, WD4, and WD5; and 5 sampling sites at Danku reservoir in dry season (DD): DD1, DD2, DD3, DD4, and DD5).

The relative abundance histogram of the top ten phyla and genera was plotted to identify the most prevalent phylum and genus in DH, DD, WH, and WD samples (Figure 3A,B). Overall, the top three abundant phyla were Proteobacteria, Actinobacteria, and Firmicutes in DH, DD, WH, and WD sampling sites (Figure 3A). The phylum Proteobacteria was more prevalent in wet season sampling sites than dry season sampling sites (WH: 69.55% vs. DH: 46.53% and WD: 84.06% vs. DD: 46.22%). Unexpectedly, Actinobacteria was more prevalent in dry season sampling sites than wet season sampling sites (WH: 1.98% vs. DH: 27.03% and WD: 1.34% vs. DD: 33.04%). Firmicutes were more prevalent in wet season sampling sites than dry season sampling sites (WH: 26.99% vs. DH: 1.09% and DH: 12.65% vs. DD: 0.45%). Further, the Kruskal–Wallis (KW) test was applied to validate the significant differences between highly abundant phyla at different sampling sites which differed significantly (*p* < 0.05) (Figure S3A). At the genus level, overall, the top abundant bacterial genera were Acinetobacter, Exiguobacterium, and Planomicrobium in wet season sampling sites (WH and WD), whereas unclassified bacteria, others, and polynucleobacter were more abundant in dry season sampling sites (DH and DD) (Figure 3B). The genus Acinetobacter was more prevalent in wet season sampling sites than dry season sampling sites (WH: 63.23% vs. DH: 0.25% and WD: 76.68% vs. DD: 0.05%). Exiguobacterium was more predominant in wet season sampling sites than dry season sampling sites (WH: 12.44% vs. DH: 0.0029% and WD: 0.76% vs. DD: 0.0011%). Similarly, Planomicrobium was also more predominant in wet season sampling sites than dry season sampling sites (WH: 3.85% vs. DH: 0.0013% and WD: 3.78% vs. DD: 0.00012%). On the other hand, polynucleobacter was more predominant in dry season sampling sites than wet season sampling sites (WH: 0.046% vs. DH: 1.74% and WD: 0.16% vs. DD: 1.04%). More than 85% of the genera in the dry season were in the unclassified category, overall. Further, the Kruskal–Wallis (KW) test revealed significant differences (*p* < 0.05) among the top abundant genera at different sampling sites (Figure S3B).

### 3.4. Beta Diversity Analysis

**Figure 4:**
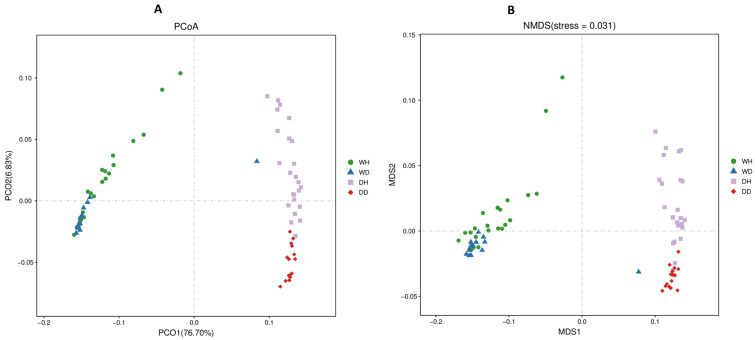
(**A**) PCoA and (**B**) NMDS analyses showing the spatiotemporal differences in the bacterioplankton communities of WH, WD, DH, and DD samples of the Danjiangkou reservoir (8 sampling sites at Hanku reservoir in wet season (WH): WH1, WH2, WH3, WH4, WH5, WH6, WH7, and WH8; 8 sampling sites at Hanku reservoir in dry season (DH): DH1, DH2, DH3, DH4, DH5, DH6, DH7, and DH8; 5 sampling sites at Danku reservoir in wet season (WD): WD1, WD2, WD3, WD4, and WD5; and 5 sampling sites at Danku reservoir in dry season (DD): DD1, DD2, DD3, DD4, and DD5).

Principal coordinate analysis (PCoA) explained the bacterioplankton communities’ spatiotemporal patterns across all sampling sites (DH, DD, WH, and WD) (Figure 4A). The total variation described by first (PCO1) and second (PCO2) components was 76.70% and 6.83%, respectively. In PCoA analysis, the bacterial communities’ composition depends on the distance between samples. The PCoA plot showed that the samples from different seasons differed significantly (*p* < 0.05) as they were separated apart (WH vs. DH and WD vs. DD). Spatially, within the same season, the wet season samples (WH vs. WD) showed more similarities than the dry season samples (DH vs. DD). Moreover, the Non-Metric Multi-Dimensional Scaling (NDMS) analysis also validated bacterioplankton spatiotemporal variations among the different sampling sites (Figure 4B). These results showed that the bacterioplankton communities’ composition was considerably more influenced by temporal (seasonal) fluctuation than spatial changes in all sampling sites.

### 3.5. Correlation Analysis between Bacterial Communities and Environmental Parameters

**Figure 5:**
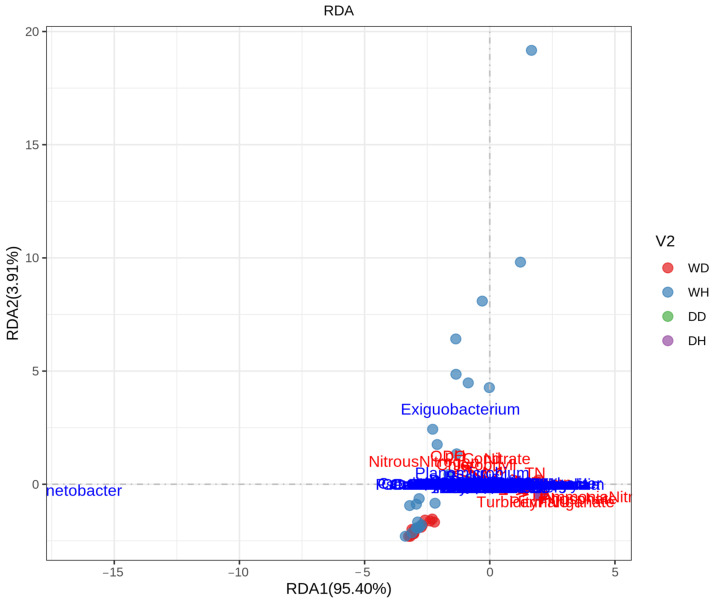
Redundancy analysis (RDA) of the bacterioplankton communities (top-ranked genera) and environmental variables in WH, WD, DH, and DD samples of the Danjiankou reservoir (8 sampling sites at Hanku reservoir in wet season (WH): WH1, WH2, WH3, WH4, WH5, WH6, WH7, and WH8; 8 sampling sites at Hanku reservoir in dry season (DH): DH1, DH2, DH3, DH4, DH5, DH6, DH7, and DH8; 5 sampling sites at Danku reservoir in wet season (WD): WD1, WD2, WD3, WD4, and WD5; and 5 sampling sites at Danku reservoir in dry season (DD): DD1, DD2, DD3, DD4, and DD5).

The relationship between bacterioplankton communities (top-ranked genera) and environmental factors during wet (WH and WD) and dry (DH and DD) seasons was explored by employing redundancy analysis (RDA) (Figure 5). The two RDA axis, RDA1 and RDA2, explained 95.40% and 3.91% of the total variance, respectively, for bacterioplankton communities. In this study, 13 environmental variables, including conductivity (Cond), optical dissolved oxygen (ODO), turbidity, pH, temperature, ammonia nitrogen, nitrate, nitrous nitrogen, total nitrogen (TN), phosphate, total phosphate (TP), chlorophyll, and permanganate, were evaluated regarding their relationship with the bacterial communities. Results showed that all these physicochemical parameters affected the dry season sampling sites’ (DH and DD) more than wet season sampling sites’ (WH and WD) bacterioplankton communities. Further, among the top-ranked genera, Planomicrobium was influenced more by these physicochemical parameters than the Acinetobacter, Exiguobacterium.

### 3.6. Bacterioplankton Functional Prediction

**Figure 6:**
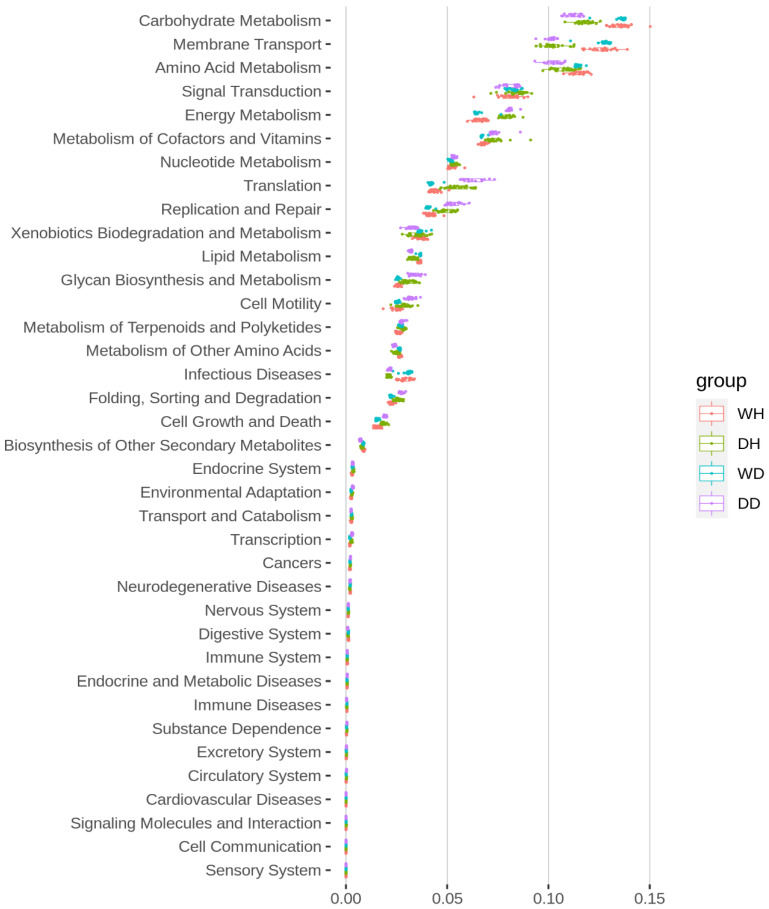
Kruskal boxplot showing the significant differences in the metabolic functional predictions of bacterioplankton communities in WH, WD, DH, and DD samples from the Danjiangkou reservoir (8 sampling sites at Hanku reservoir in wet season (WH): WH1, WH2, WH3, WH4, WH5, WH6, WH7, and WH8; 8 sampling sites at Hanku reservoir in dry season (DH): DH1, DH2, DH3, DH4, DH5, DH6, DH7, and DH8; 5 sampling sites at Danku reservoir in wet season (WD): WD1, WD2, WD3, WD4, and WD5; and 5 sampling sites at Danku reservoir in dry season (DD): DD1, DD2, DD3, DD4, and DD5).

The sequencing data from the 16S rRNA gene were used to predict the functional categories of bacterioplankton communities in all samples (DH, DD, WH, and WD) using the Tax4Fun package (Figure 6). Results displayed that the important top five enriched functions were carbohydrate metabolism, membrane transport, amino acid metabolism, signal transduction, and energy metabolism in all sampling sites. Carbohydrate metabolism, membrane transport, and amino acid metabolism functions were significantly higher (*p* < 0.05) during the wet season (WH and WD) compared to the dry season (DH and DD). In contrast, signal transduction and energy metabolism were significantly higher (*p* < 0.05) in the dry season (DH and DD) compared to the wet season (WH and WD). Overall, seasonal variations significantly affected the functional features of bacterioplankton communities in all samples more than spatial variations. 

## 4. Discussion

China has a severe shortage of water resources, as well as significant seasonal and regional variations in the allocation of water resources [27]. In north China, more than 300 cities are reported to have insufficient water supply, as recently identified by the National Water Resource Report. To alleviate the water shortage problems in China, the longest water diversion project, MR-WSNDP, was initiated in 2014. For this project, the Danjiangkou Reservoir’s average water storage level, which serves as a water supply for the MR-SNWDP, was increased from 157 to 170 m. However, the water transfer has interrupted the bacterioplankton communities in the reservoir in some ways [28]. Bacterioplankton play a significant role in various geochemical cycles and in the transport of carbon to the microbial food web occurring in aquatic ecosystems such as rivers, water channels, reservoirs, and lakes [29,30]. Bacterioplankton communities’ compositions can serve as indicators of aquatic environmental conditions since they are typically responsive to environmental instabilities and water quality [15,19,31,32,33]. Studies revealed that the bacterioplankton communities’ compositions can be influenced in response to spatiotemporal and environmental changes [30,34]. Therefore, it is necessary to monitor the spatial and temporal distribution of bacterioplankton communities to understand the dynamics of the reservoir [15,28,30]. So far, significant spatiotemporal fluctuations in bacterioplankton communities’ abundance and diversity have been recorded in various aquatic ecosystems [35,36,37].

Recently, advancements in next-generation sequencing techniques have revolutionized microbial studies in terms of their diversity, structure, richness, and reaction to environmental variability [38,39]. In the current study, 16S rRNA gene V4 region sequencing was conducted using the Illumina PE250 platform to analyze the bacterioplankton communities’ composition at different sampling points in Hanku and Danku reservoirs during dry and wet seasons. The sequencing results showed the highly dynamic and complex environment of the Danjiangkou Reservoir. The ACE and Shannon indices revealed significant spatiotemporal differences in the bacterioplankton composition in the Danjiangkou Reservoir. Results showed that bacterioplankton community abundance and diversity were higher during the dry season as compared to the wet season at both sampling sites. Further, the ACE and Shannon indices exhibited that the order of bacterioplankton richness and diversity was DH > DD during the dry season and WH > WD during the wet season. In previous studies, the abundance and diversity indices were higher during summer compared with the spring season [30]. The bacterioplankton community predominantly comprised three phyla Proteobacteria, Actinobacteria, and Firmicutes in all samples (DH, DD, WH, and WD). However, Acinetobacter, Exiguobacterium, and Planomicrobium were the dominant genera in WH and WD samples, while unclassified and other genera were abundant in DH and DD samples. In previous studies, these dominant phyla and genera have also displayed significant seasonal and spatial differences in various aquatic environments and their potential association with the water quality [28,30,32,40]. Furthermore, studies have also reported good water quality during the dry season as compared with the wet season [1,3,41]. Proteobacteria were the dominant constituent of the bacterial community and were found to be consistent in preceding studies [42,43,44]. Proteobacteria serve as a potential water quality indicator as they are positively correlated with heavy metals pollution. The relatively high abundance of Proteobacteria indicated poor water quality during the wet season, as already reported [45]. Proteobacteria are involved in various nitrogen cycles that produce nitrogenous nutrients for phytoplankton growth [46,47]. Higher amount of these nitrogenous compounds may reflect the poor water quality [3]. Exiguobacterium and Planomicrobium are potentially involved in various sulfur-denitrification processes [48], and are negatively correlated with TN and phosphorus [49]. Firmicutes play an important role in degrading either simple or complex organic molecules such as cellulose, and lactic acid into pyruvate and acetyl coenzyme A, resulting in the production of metabolites such as methane and ethanol [44,50]. Additionally, Firmicutes have also been found to be the most abundant microbe in rivers and have served as an effective indicator for fecal pollution [51]. Further, a Kruskal–Wallis (KW) test showed significant differences between the top-ranked phyla and genera bacterioplankton communities during both seasons. Moreover, regarding beta diversity, PCoA and NDMS showed that seasonal variations had more influence on community composition than the different sampling sites. These results were consistent with earlier research, where the seasonal variations highly affected the bacterial community composition more than the spatial variations [28,35]. 

In this study, the environmental factors varied in different seasons and sampling sites and showed a differential effect on the bacterioplankton communities. The RDA analysis showed that 13 environmental variables, including conductivity, optical dissolved oxygen, turbidity, pH, temperature, ammonia nitrogen, nitrate, nitrous nitrogen, total nitrogen (TN), phosphate, total phosphate, (TP), chlorophyll, and permanganate, affected the bacterioplankton community composition in the Danjiangkou Reservoir. Results showed that these variables highly influenced the bacterioplankton communities during the dry season (DH and DD) as compared to the wet season (WH and WD). This is consistent with the higher bacterioplankton diversity in the dry season than in the wet season, as shown by diversity analysis. Further, among the top three genera, Planomicrobium was more highly influenced by these physicochemical parameters than Acinetobacter and Exiguobacterium. Planomicrobium is involved in various N-cycles and removes cyanobacterial blooms by decreasing NH_4_-N, thus negatively correlated with TN, N:P ratio, and nitrogenous compounds and positively correlated with DO [3,47]. Further, a high abundance of Acinetobacter during the wet season reflected poor water quality in prior studies [52]. The high abundance of Acinetobacter during wet season could be a result of non-treated discharges from wastewater treatment plants [52]. Earlier studies revealed nitrogen and phosphorus as important variables affecting the composition of bacterial communities in the DJR [18,19,53]. Additionally, Polynucleobacter was more abundant in dry season sampling sites than wet season sampling sites. Previous studies revealed that environmental variables including pH and temperature are more likely to influence Polynucleobacter community composition in an aquatic environment [54]. In previous studies, Polynucleobacter abundance was similar between seasons and across the sampling sites [55]. Polynucleobacter has the ability to biodegrade pesticide chemicals found in freshwater after use in agriculture, which improves the water quality [56]. Dissolved oxygen (DO) concentration is an important factor reflecting water quality as it significantly affects microbial composition, as reported in previous studies [57,58]. Studies have proven that seasonal changes in environmental variables can majorly affect bacterial community composition and its impact on water quality [59,60,61]. Further, the functional predictions of metabolic pathways for bacterioplankton communities were analyzed, which revealed that the top five functions were carbohydrate metabolism, membrane transport, amino acid metabolism, signal transduction, and energy metabolism. Results showed that carbohydrate metabolism, membrane transport, and amino acid metabolism were significantly higher in WH and WD samples. Previous studies also showed the high abundance of these metabolic functions of bacterial communities in aquatic ecosystems [62]. Carbohydrate and amino acid metabolism play a vital role in the survival of bacterial communities by utilizing substrates in aquatic environments, while membrane transport is crucial for carbon fixation [63]. It can be inferred from our findings that spatiotemporal variations had significant effects on bacterioplankton communities’ composition and functions, influenced by environmental variables. Further, a higher abundance of certain bacteria such as Acinetobacter significantly affected the water quality during the wet season compared with the dry season.

## 5. Conclusions

The present study concluded that seasonality significantly impacts bacterioplankton communities, and the dry season had more diverse bacterial communities influenced by environmental parameters. Certain bacteria such as Acinetobacter deteriorated the water quality during the wet season compared with the dry season. Our findings also provide evidence and insights about the effects of environmental changes on bacterioplankton communities and their functions. Further, bacterioplankton communities and their relationships with environmental parameters are driving factors for water quality assessment. It is recommended to conduct continuous monitoring of water quality in the DJR over a prolonged period to track any changes in water quality trends. However, further studies are warranted to elucidate the nature of seasonal effects on individual microbial communities and their subsequent effects on aquatic ecosystems. Our findings have significant implications for water resource management in China, and other countries facing similar challenges. 

## Data Availability

Data supporting reported results are available as supplementary files and provided on reasonable request.

## Supplementary Materials

The following supporting information can be downloaded at: https://www.mdpi.com/article/10.3390/life13051206/s1, Figure S1: Venn diagram showing the observed OTUs numbers that are unique and shared among DH, DD, WH, and WD samples; Figure S2: (A) Rarefaction and (B) rank abundance curves of observed OTUs in DH, DD, WH, and WD samples; Figure S3: Kruskal boxplot showing the significant differences in the relative abundance of bacterioplankton communities in DH, DD, WH, and WD samples at (A) phylum and (B) genus levels in the Danjiangkou reservoir; Table S1: Detailed sampling information with complete labels.
