# Supplementary material for: Dynamics of Bacterioplankton Communities during Wet and Dry Seasons in the Danjiangkou Reservoir in Hubei, China
Source: Life (Basel). 2023 May 17;13(5):1206. doi: 10.3390/life13051206 (PMC10220977; doi:10.3390/life13051206)
Supplement: Supplementary file 1 [file life-13-01206-s001.zip › life-2346762-supplementary.pdf]

life

MDPI

Supplementary material

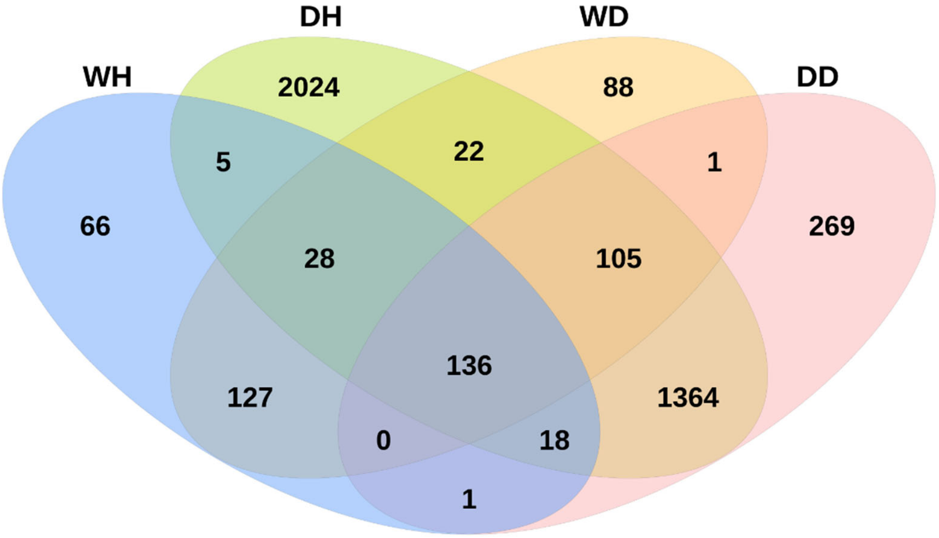

Figure S1. Venn diagram showing the observed OTUs numbers that are unique and shared among DH, DD, WH, and WD samples.

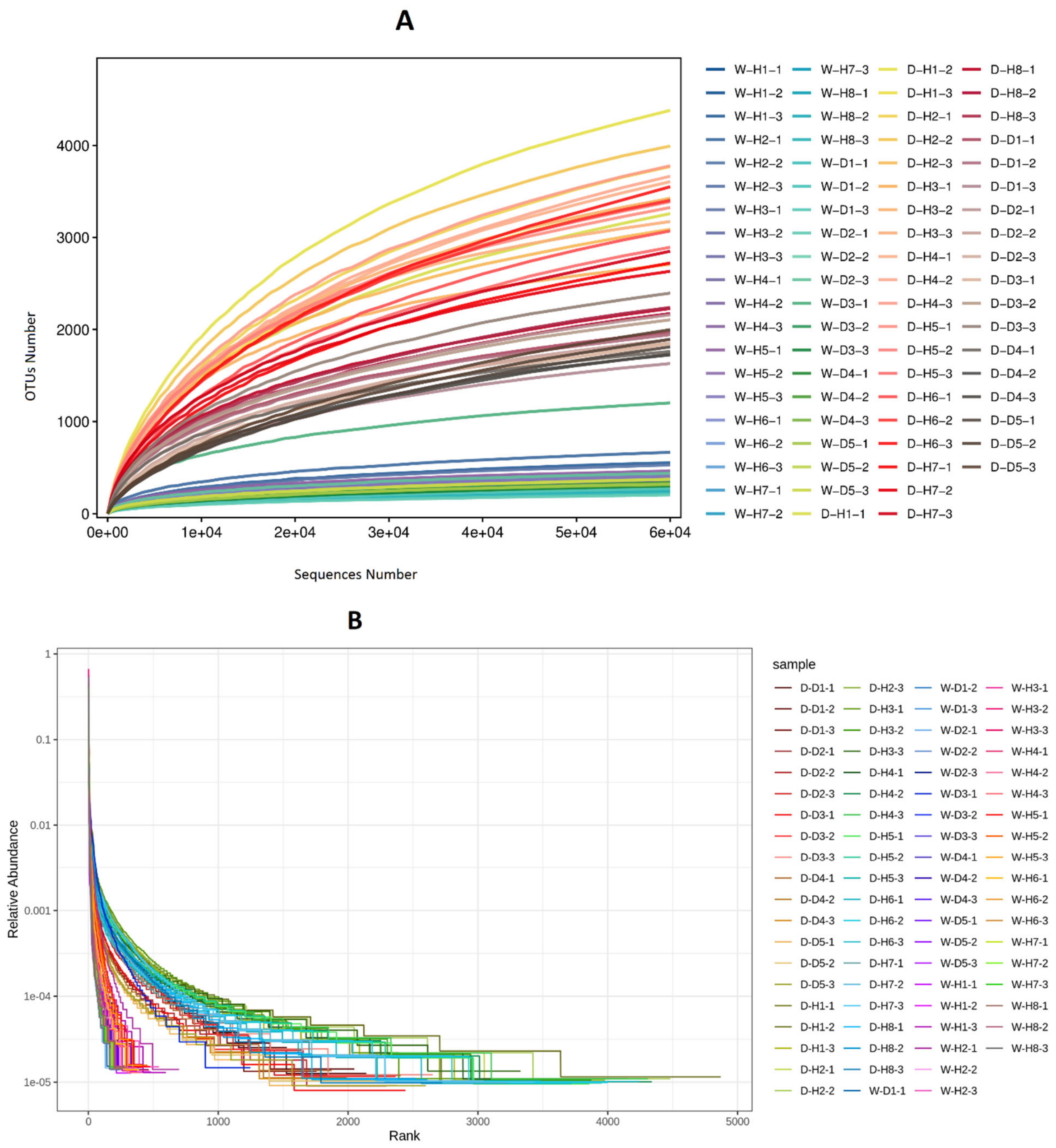

Figure S2. (**A**) Rarefaction and (**B**) rank abundance curves of observed OTUs in DH, DD, WH, and WD samples.

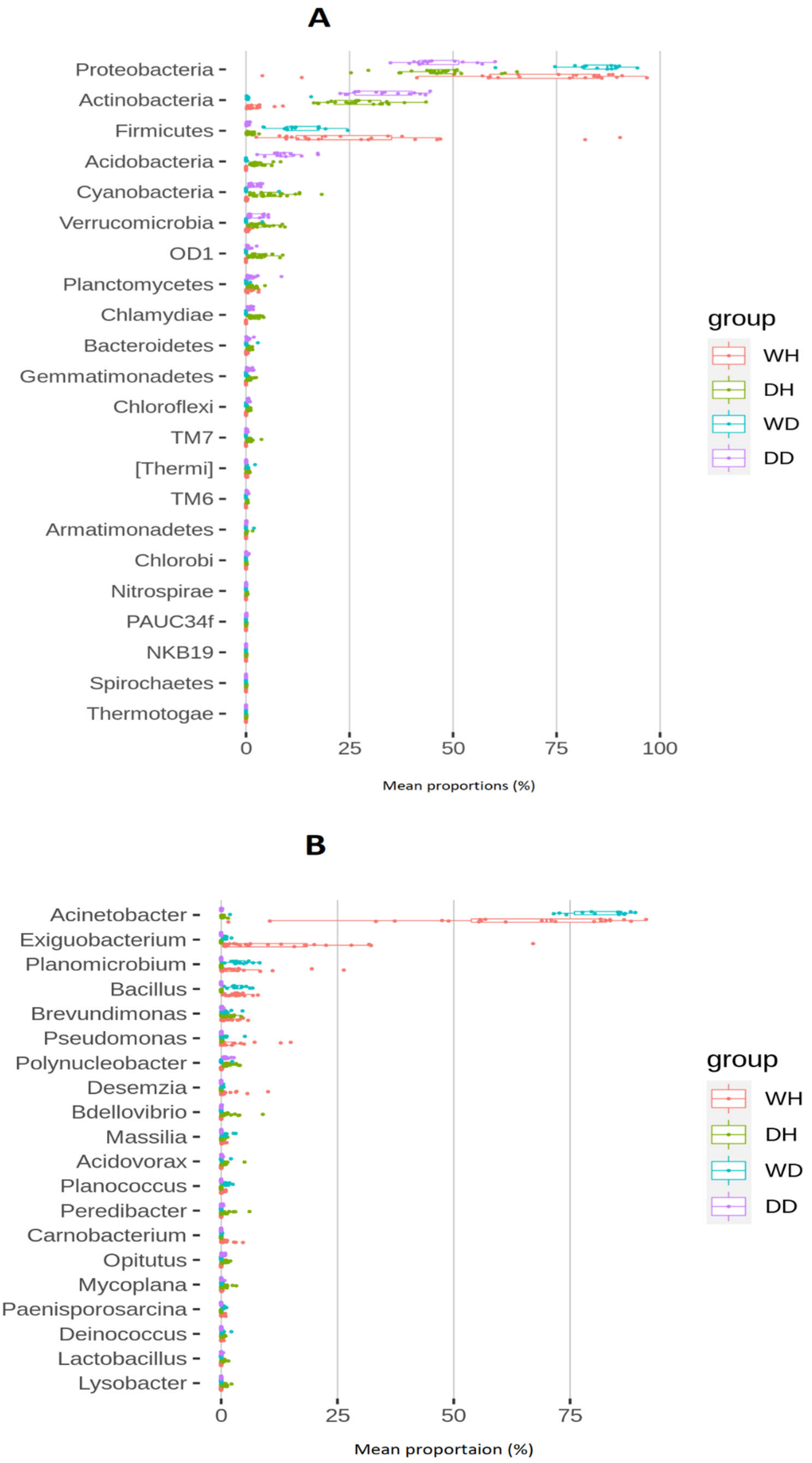

Figure S3. Kruskal boxplot showing the significant differences in the relative abundance of bacterioplankton communities in DH, DD, WH, and WD samples at (**A**) phylum and (**B**) genus levels in the Danjiangkou reservoir.

Table S1. Detailed sampling information with complete labels.

Season	Sampling Site	Samples	Samples with Replicates
Wet Season (W)	Hanku (WH)	WH1	W-H1-1<br>W-H1-2<br>W-H1-3
		WH2	W-H2-1<br>W-H2-2<br>W-H2-3
		WH3	W-H3-1<br>W-H3-2<br>W-H3-3
		WH4	W-H4-1<br>W-H4-2<br>W-H4-3
		WH5	W-H5-1<br>W-H5-2<br>W-H5-3
		WH6	W-H6-1<br>W-H6-2<br>W-H6-3
		WH7	W-H7-1<br>W-H7-2<br>W-H7-3
		WH8	W-H8-1<br>W-H8-2<br>W-H8-3
	Danku (WD)	WD1	W-D1-1<br>W-D1-2<br>W-D1-3
		WD2	W-D2-1<br>W-D2-2<br>W-D2-3
		WD3	W-D3-1<br>W-D3-2<br>W-D3-3
		WD4	W-D4-1<br>W-D4-2<br>W-D4-3
		WD5	W-D5-1<br>W-D5-2<br>W-D5-3
Dry season (D)	Hanku (DH)	DH1	D-H1-1<br>D-H1-2<br>D-H1-3
		DH2	D-H2-1<br>D-H2-2<br>D-H2-3
		DH3	D-H3-1<br>D-H3-2

			D-H3-3
		DH4	D-H4-1
			D-H4-2
			D-H4-3
		DH5	D-H5-1
			D-H5-2
			D-H5-3
		DH6	D-H6-1
			D-H6-2
			D-H6-3
		DH7	D-H7-1
			D-H7-2
			D-H7-3
		DH8	D-H8-1
			D-H8-2
			D-H8-3
	Danku (DD)	DD1	D-D1-1
			D-D1-2
			D-D1-3
		DD2	D-D2-1
			D-D2-2
			D-D2-3
		DD3	D-D3-1
			D-D3-2
			D-D3-3
		DD4	D-D4-1
			D-D4-2
			D-D4-3
		DD5	D-D5-1
			D-D5-2
			D-D5-3
